# Acute small bowel obstruction caused by a fan-shaped congenital band in a child: a case report

**DOI:** 10.3389/fped.2025.1539677

**Published:** 2025-03-11

**Authors:** Qingnan Lan, Jianhua Zhong, Yi Wang, Haiwei Zhu, Xin Liu, Yanping Guo, Zhibo Qu

**Affiliations:** Department of Pediatric Surgery, Dongguan Children’s Hospital, Dongguan, China

**Keywords:** wide fan-shaped, congenital band, small bowel obstruction, pediatric surgery, case report

## Abstract

Congenital bands are rare abnormal structures that can occur anywhere in the gastrointestinal tract, and intestinal obstruction caused by these bands is uncommon in children. To our knowledge, there have been no reported cases of small bowel obstruction (SBO) resulting from a wide, fan-shaped congenital band located on the surface of the ileum and mesentery. We present the case of a 13-year-old girl who developed SBO due to a unique fan-shaped band that compressed a segment of the ileum. The diagnosis of intestinal obstruction was confirmed through x-ray and CT imaging, and the location and cause of the obstruction were further elucidated during exploratory laparotomy. The band was excised, with no bowel resections required. Congenital or spontaneous bands are rare causes of bowel obstruction, and accurately diagnosing this condition prior to surgery can be challenging. When considering the potential causes of intestinal obstruction, it is crucial to include congenital bands in the differential diagnosis.

## 1 Introduction

Adhesive SBO is a common surgical condition worldwide, most often resulting from previous abdominal surgery (1). However, cases of adhesive SBO occurring without a history of abdominal surgery or trauma are rare, and congenital bands are an even more uncommon cause. These congenital bands are most commonly found in the ileum, followed by the colon, mesentery, omentum, peritoneum, and jejunum (2). Congenital bands can cause mechanical obstruction, often necessitating surgical intervention (3). To date, approximately 51 cases of congenital bands in children have been reported in the literature. Notably, none of these cases describe scallop-shaped congenital bands covering the ileum and its mesenteric surface (4).

## 2 Case presentation

A 13-year-old girl was transferred to our hospital after experiencing recurrent abdominal pain for two years and worsening abdominal pain accompanied by vomiting for three days. She was diagnosed with gastroenteritis three days earlier at a local hospital and received anti-infective treatment. However, the patient was transferred to our hospital due to the continued worsening of abdominal pain and the cessation of bowel movements and passing of gas for one day.Medical history: The patient had a history of recurrent, unexplained abdominal pain, which was not localized to a specific area and usually resolved within 3–4 h after each episode. These episodes occurred irregularly. She had no history of abdominal surgery, abdominal trauma, gastrointestinal bleeding, or peritonitis. On physical examination, the patient was in a flexed, passive position, with diffuse tenderness and rejection of palpation throughout the entire abdomen,but no rebound tenderness or abdominal muscle rigidity was noted. No significant masses were palpated. Laboratory results showed a white blood cell (WBC) count of 12.63 × 10^9^/L and a C-reactive protein (CRP) level of 75 mg/L.

**Figure 1:**
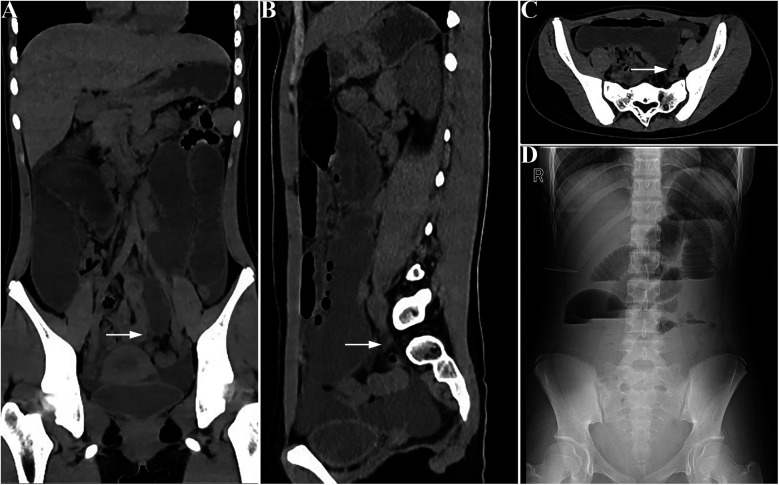
Preoperative presentation: CT imaging reveals significant distension and fluid accumulation in the small intestine, with the distal colon appearing collapsed. **(A)** coronal view, **(B)** sagittal view, **(C)** cross-sectional view; the white arrow indicates the obstruction point). Panel **(D)** shows an abdominal upright x-ray following preoperative conservative treatment, demonstrating dilated small bowel loops.

**Figure 2:**
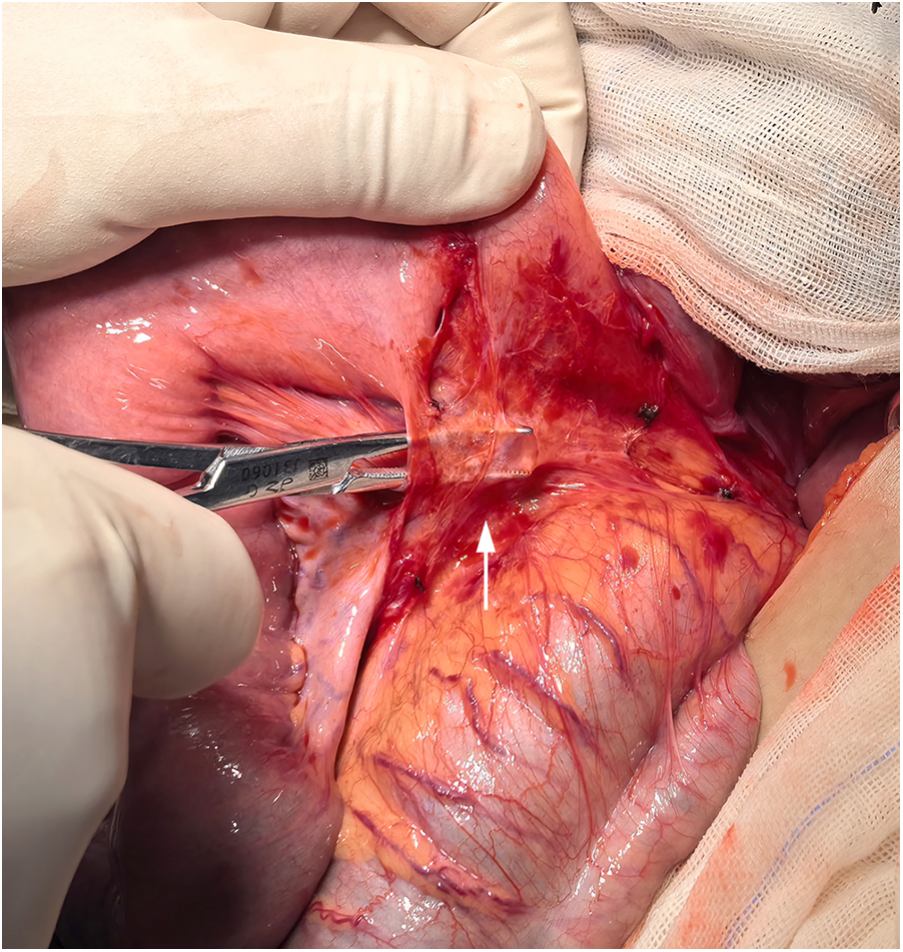
A fan-shaped congenital band was observed covering the surface of the ileum during surgery.

Abdominal CT scans revealed significant fluid accumulation and distension of the small intestine, along with hollowing of the distal colon (Figures 1A–C), confirming the diagnosis of intestinal obstruction. Abdominal pain persists and worsens despite fasting and continuous gastrointestinal decompression, the patient's symptoms persisted, and a follow-up abdominal x-ray showed no improvement in the obstruction (Figure 1D). Consequently, a laparotomy was performed at our hospital through a transverse incision in the right lower abdomen. During the operation, a wide band was identified connecting the root of the mesentery to the ileocecal wall, approximately 30 cm from the ileocecal junction (Figure 2). The band tightly encircled both the ileocecal wall and the corresponding mesentery in a fan-shaped pattern. This band was shorter than the mesentery, and due to its tension and compression, the segment of the intestine attached to the band was pulled toward the posterior abdominal wall, resulting in restricted mobility. The proximal small intestine was markedly dilated and congested, while the distal bowel appeared empty. The band was divided using electrical coagulation near the ileum, thereby releasing the compressed intestinal segment. The abdominal cavity was then closed following the placement of drainage.The patient had a bowel movement on the same day after the surgery, indicating an improvement in their condition. The patient was discharged 6 days postoperatively. No recurrence of symptoms was observed during subsequent follow-up. The excised band was confirmed to be fibrous by final pathological examination.

## 3 Discussion

Intestinal obstruction caused by congenital bands is extremely rare in both adults and children. These bands are often difficult to classify and define (5, 6). Congenital bands account for approximately 3% of cases of bowel obstruction, almost always involving the small bowel (7). Due to their low incidence, it is challenging to consider this etiology in patients presenting with ileus in clinical practice.

Congenital bands can occur in various parts of the gastrointestinal tract, including the colon, mesentery, omentum, and jejunum; however, the most common location is around the terminal ileum (2, 8). At present, there are few literatures reporting such cases, and the etiology of congenital band formation is not clear. Yang et al. suggested that congenital bands develop during embryogenesis due to abnormal adhesion of peritoneal folds, potentially caused by infectious or ischemic events. Although congenital in nature, these bands typically present in childhood, but they can also cause SBO at any age (8).

Currently, most congenital bands reported in the literature are cable-like structures; however, wide, fan-shaped congenital bands have not been previously documented. We analyzed the recurrent abdominal pain in this case over the past two years and found it to be associated with band compression. In most cases, the proximal intestinal contents gradually pass through the obstructed segment due to differences in intestinal pressure and peristalsis. As a result, abdominal pain is often self-limiting. Progression to complete obstruction may occur following a gastrointestinal infection in the early stages, leading to disruption of intestinal peristalsis or transient paralysis. This causes accumulation of intestinal contents at the proximal end of the band, which, due to the effect of gravity, results in the formation of an acute angle on both sides of the band. The continued accumulation of intestinal contents exacerbates the angle, further narrowing the obstruction and creating a vicious cycle that leads to complete obstruction. This process differs from previously reported cases of intestinal obstruction caused solely by band compression.

In general, the bands directly compress the intestines or cause the intestinal loops to enter the “snare” between the band and the mesentery or intestinal wall, leading to an internal hernia and intestinal obstruction. This often presents with typical clinical symptoms and signs of mechanical intestinal obstruction, including abdominal cramps, nausea, bloating, with or without vomiting, and intractable constipation (6). Physical examination is crucial for identifying signs of strangulation and ruling out abdominal wall or inguinal hernias (9). Signs of strangulation include persistent, severe pain, tenderness, and reduced peristalsis or abdominal silence (2).

Laboratory studies, such as elevated WBC count and CRP levels, can provide some indication of peritonitis, although the sensitivity and specificity of these tests are relatively low. The sensitivity of plain x-rays for diagnosing SBO is approximately 70%. Typical radiographic features include dilation of the small bowel loops, multiple air-fluid levels, and an empty colon. Additionally, standing abdominal radiographs (plain abdominal x-rays) may reveal pneumoperitoneum secondary to intestinal perforation (10). CT scan has irreplaceable value in confirming the diagnosis, location, and level of small bowel obstruction, accurately differentiating the different causes of small bowel obstruction by excluding other potential conditions. It demonstrates approximately 90% accuracy in predicting strangulation and the need for emergency surgery. Additionally, CT imaging reveals the distinctive features of closed-loop small bowel obstruction and ischemia (9, 11, 12). Therefore, the preoperative identification of congenital bands as the cause of SBO is often an exclusionary diagnosis and must be based on evidence that there is no other cause of obstruction.

The primary treatment for intestinal obstruction caused by congenital bands is surgery, which is essential for both confirming the diagnosis and providing definitive treatment (12). Laparoscopic exploration has gained popularity in recent years and has been shown to be both safe and feasible for diagnosing and treating adhesive band-related SBO, particularly in cases involving limited intestinal distention and a single band (9, 13). According to the 2017 World Society of Emergency Surgery guidelines (9), laparoscopy is indicated for cases of single adhesive obstruction with mild intestinal dilation. In this case, due to CT findings demonstrating severe intestinal dilation (diameter > 4 cm) and complex anatomical structures, open surgery was selected. The advantages of laparoscopic exploration include reduced trauma and a lower incidence of major postoperative complications (14, 15). If bowel necrosis is detected or the site of obstruction cannot be identified during laparoscopy, a laparotomy should be performed to remove the band and, if necessary, resect the necrotic bowel (7).

## Data Availability

The original contributions presented in the study are included in the article/Supplementary Material, further inquiries can be directed to the corresponding author.
